# Work/Life Relationships and Communication Ethics: An Exploratory Examination

**DOI:** 10.3390/bs12040104

**Published:** 2022-04-11

**Authors:** Janie M. Harden Fritz

**Affiliations:** Department of Communication & Rhetorical Studies, Duquesne University, Pittsburgh, PA 15282, USA; harden@duq.edu

**Keywords:** personal workplace relationships, workplace romance, workplace friendships, work/life, communication ethics

## Abstract

Workplace relationships that transcend formal role boundaries offer benefits and challenges to organizations and relational participants. Communicative processes that form and maintain these relationships can be examined from a communication ethics perspective focused on the outcomes emerging from these relationships that define particular goods for personal and organizational life. The blended nature of these relationships makes them host to potentially competing goods tied to public and private concerns. Considering the connection of virtue approaches to communication ethics in organizational settings to the turn to positive approaches to communication and organizational theory reveals avenues for ethical reflection and action in these increasingly important relational forms.

## 1. Introduction

Communicative interactions between and among employees are vital to the success of organizations—indeed, communicative work constitutes organizations [1]. Because relationships are constructed through communicative interactions [2], the communicative constitution of organizations includes relationships within its purview. As people spend more time at work, organizations provide a fruitful context for studying relationship constitution and ongoing development [3]. Some of those relationships will remain at a level marked by professional distance, while others will develop into close relationships [4]. Whatever trajectory organizational relationships follow, organizations and relationships will be mutually constituted, beginning with the organizational socialization process [5].

The study of workplace relationships integrates two significant areas of the communication field, organizational communication and interpersonal communication, and contributes to the interdisciplinary domain of personal and social relationships. Personal relationships in the workplace, those moving beyond the formal, role-bound elements of connection defined by organizational structure and characterized by varying degrees of closeness or intimacy [3], constitute a specific focus of study with an identifiable and growing body of literature. Understanding this relational form is important because of the significant outcomes associated with these types of relationships [3].

Participation in organizations involves significant choices about communicative behavior; these communicative choices are valenced, protecting and promoting some good [6]. Personal relationships in the workplace hold implications for the wellbeing not just of the parties involved, but of other organizational members [3], the organization, and its other stakeholders as well [7,8]. More specifically, because communication in the development and maintenance of personal workplace relationships involves choice [9,10] on the part of relational partners; holds consequences for others, including the organization [3]; and can be assessed according to standards of right and wrong, even if the basis for those standards is not always articulated or made explicit [11], workplace relationships are an important context for communication ethics [7,8,12,13,14,15,16].

The increased attention to organizational ethics, missions, and values [17,18,19,20] suggests the value of workplace relationships as a site of ethical engagement. Bridge and Baxter’s [21] study of dialectical tensions in work relationships points to ethical issues in terms of the good of each person and the organization, as does the work of Conrad and Poole [22] in their acknowledgment of tensions between person and organization. The research streams of multiple scholars (see Horan et al. [3] for a review) likewise point to issues valuable for ethical considerations. Although virtue and value structures vary considerably across persons and organizations [6], issues of consequences, responsibility, and harm emerge in every organizational context. Organizational incivility [23,24,25] and other “dark side”—or, alternatively, “inappropriate,” following Duck and VanderVoort’s [26] terminology, as suggested by Horan, Chory, Craw, and Jones [3] in their thoughtful response to Long’s [27] concerns about connotations of the term “dark side”—communication issues in the workplace [28] point to relationships that result in distress or harm to others, placing these areas of study within the realm of communication ethics.

The intersection of organizational ethics, organizational communication ethics, and interpersonal communication ethics finds its central point in the area of personal work relationships, blended relationships [3,21] with elements of both public roles and private associations. One of the greatest concerns prompting considerations of communication ethics in work relationships is related to this overlap of the private and public spheres, which can result in unintended consequences, as observed by Ashcraft [29]. In the work relationship literature, this concern has been investigated within the framework of work/life boundary management [3].

These considerations prompt a foundational question for communication ethics in workplace relationships: what are the conditions under which personal relationships in the workplace can protect and promote the organization, the parties to the relationship, the relationship itself, the organization’s mission, and other goods in the organizational context? Workplace relationships could be considered to operate within the boundaries of communication ethics when their communicative practices support the work of the organization and the organization itself, the lived experience of work of each party to the relationship, and others in the workplace [8]. Beyond those considerations, the horizons for ethical behavior may be quite broad. The framework for ethics one engages will point to reasons other than idiosyncratic personal preference for approaching personal relationships in organizations. Utilitarian, deontological, narrative, dialogic, feminist, and other approaches to communication ethics suggest different foci for attentiveness [6]; in the context of organizations, these ethical systems provide insights for workplace relationships [12]. This article offers a representative review of communication ethics relevant to workplace relationships, reflects on some considerations regarding communication ethics in workplace relationships, and suggests future directions. The next section, Section 2, explores the development of interest in communication ethics and interpersonal communication in workplace relationships, highlighting virtue ethics as the eventual focus of work in this area.

## 2. Communication Ethics as an Area of Study

The appearance of the first handbook of communication ethics [30], with a second edition forthcoming, marks the importance of communication ethics as an area of study, as it intersects with multiple other subfields in the broad communication discipline. Duck [31] notes the cyclical nature of academic research, including the study of workplace relationships, identifying the turn to positive relationships in the workplace as part of that pattern. Each time the pendulum moves back to an original starting point, the returning point is different, however. New findings have added texture and insight to enduring themes such as the relationship between attraction and similarity, patterns of love, and the role of attachment in relationships [31]. Duck also observes that disciplinary areas do not always take each other’s work into account, resulting in findings across related areas (such as management studies and organizational communication) that could inform one another, but often do not. This trend toward fragmentation can be seen in the 20th century history of communication within the multi-faceted, broad field itself [32].

The current focus on communication ethics emerges at the intersection of several strands of the communication field that were viewed as separate and of very different institutional value [32]. According to one treatment, this history reveals a division between the social scientific and rhetorical domains, in which the study of communication ethics was relegated to the rhetorical area [32], virtue-related terms were translated into mental hygiene terms, and the good of society became the goal of communicative practice in an era of “social integration” ([33], p. 111). As a social science approach to interpersonal communication research gained prominence, a commitment to the good of persons and relationships, although not grounded explicitly in a philosophical tradition, guided this developing area [11].

Over the last decades of the 20th century, communication ethics and philosophical dialogue became integrated with what is now known as philosophy of communication [11]. Arnett’s work offered phenomenological dialogue as a way to situate the self within larger meaning structures, narratives that guide action [11,34]. Differences between psychological approaches to dialogue and philosophical approaches to dialogue reveal a division between two different strands of research relevant to communication ethics and to interpersonal communication—and, by extension, to organizational communication.

The psychological approach to interpersonal dialogue rests on an implicit commitment to expressive individualism [35] or emotivism [36], ethical decision-making based on one’s personal inclinations or preferences. The philosophical approach to interpersonal dialogue offers different assumptions, grounding, or understandings of the good based on religious tradition and phenomenology [11], focusing on meaning arising between relational parties [37]. Interpersonal dialogue from a philosophical perspective is a hermeneutic process of communicative *praxis* [38], in which individual selves are not ignored but are called forth responsively in answer to situational and relational constraints, including the narrative or meaning-rich ground within which each person is situated [39]. This understanding of embeddedness is important for an understanding of workplace relationships as situated within an organization, an “Other” to whom the relationship is accountable [14]. Arnett’s [40] examination of implications of Martin Buber’s dialogue for organizational life, an application of this philosophical perspective, could be considered one of the first approaches to interpersonal communication ethics in organizations in the communication field. From this perspective, organizational communication ethics can be framed as a type of communicative praxis, or “practical philosophy [that] is grounded in a context of action for the common good” ([41], p. 211).

This path of development in the area of communication ethics ran parallel to a renewed focus on the influence of interpersonal communication on personal wellbeing, as well as on a turn to positive approaches to personal [42] and organizational [43] relationships—another shift in patterning that pushed off from the recognition that not all communication is benign or beneficial and subsequent “dark side” studies of communication and relationships [44]. This “dark side”, or inappropriate, focus emerged, as well, in the organizational behavior literature, an area rich with findings that fit squarely within the domain of communication [24,28]). Chory and Hubbell’s [45] work on organizational injustice and distrust as predictors of antisocial communication and behavior was an early indicator of the intersection of communication ethics and the problematic or inappropriate side of communication in workplace settings. Concerns about bullying [46], destructive organizational communication [47], ostracism and cliques [48,49], and related problematic organizational behaviors framed by Fritz [13,24,25,28] as communicative phenomena, such as aggression, antisocial work behavior, counterproductive work behavior, insidious workplace behavior mobbing, organizational deviance, organizational misbehavior, organizational retaliatory behavior, petty tyranny, workplace hostility, and workplace incivility, raised issues of relational communication ethics in the workplace. Fritz’s [50] treatment of bullying as unethical workplace behavior from a professional civility perspective highlights harms to multiple dimensions of organizational life, including relationships.

The next section explores positive communication and its relationship to virtue ethics.

## 3. Positive Communication and the Turn to Virtue Ethics

The shift to positive approaches to communication and relationships took form as a focus on human happiness, or the Aristotelian notion of *eudaimonia* [12], a central feature of virtue ethics—its *telos,* or aim. *Eudaimonia* as a philosophical term has its psychological counterpart in the work of Diener, Seligman, and Csikszentmihalyi on human happiness [12], perspectives that, in large part, undergird the work of positive organizational [43,51] and communication [42] scholars. From a perspective of virtue ethics in the workplace, in fact, we could make a central claim: “The underlying assumption of research on workplace relationships is that constructive interpersonal relationships are a good in, of, and for human life in organizations” ([13], p. 258). Lutgen-Sandvik, Riforgiate, and Fletcher’s [47] study of discourses of positivity in the workplace highlighted the pleasant and joyous experiences of work in contradistinction to problematic ones, highlighting the potential of workplace relationships as a source of human happiness.

Interest in virtue ethics in the communication field more generally, particularly in applied and philosophical approaches, has grown significantly over the last two decades [15]. Cheney et al. [12] situated professional life within a virtue ethics perspective, offering insights for workplace relationships as situated within an organizational setting and calling for the particularity of care as articulated by Gilligan [52]. Fritz [13] developed a professional civility framework to address relationships in organizations as part of a comprehensive treatment of communicative virtue in the workplace emerging from the history of the professions in the United States [8]. Other researchers also examined virtue ethics as an approach to organizational communication ethics (e.g., [53]), but this work was externally focused rather than directed toward relationships within organizational settings.

## 4. Organizational Communication Ethics: General Approaches

Organizational communication ethics, organizational ethics, and business ethics are related areas of study with varying degrees of overlap. Differences rest with connotations or implications of the various terms, the emphasis placed on a given area of activity addressed as part of the domain (see, for instance, [6,54]), and the degree of identification of the area as a recognized field of study. These areas’ differences and points of intersection parallel, to some degree, the kindred, yet distinctive, areas of organizational, managerial, business, and corporate communication addressed by Shelby [55] in her efforts to distinguish these areas of study.

*Organizational ethics* could be considered an umbrella term, the broadest of the three, with an emphasis on institutional culture and internal and external practices of organiza- tions considered through an ethical lens, including communication and codes of conduct within and across various organizational types and forms (e.g., [56]). *Business ethics* is a distinct field of study drawing from multiple disciplines [57]. Addressed originally through a philosophical lens [58], the theoretical and practical scope of business ethics has grown exponentially over the decades, with two journals, *Journal of Business Ethics* and *Business Ethics Quarterly*, devoted explicitly to this domain. Although it does not appear that one formal, agreed-upon definition has been identified for this established, yet still developing, area, one could say that the horizon of business ethics scholarship encompasses the ethical implications of functions and processes taking place in and emerging from environments devoted to the exchange of goods and services. *Organizational communication ethics* could be considered a subset of communication ethics (e.g., [6,16]) or a subset of organizational communication (e.g., [20,59,60]), focusing on communication taking place within, constituting, and produced by organizations and their representatives that holds ethical implications. Topics addressed in one area are often addressed in another, with the emphasis and scholarly resources brought to bear on a topic varying according to the disciplinary area of the scholar and the domain addressed. For example, organizational communication scholars might examine areas addressed by organizational and business ethics scholars through the resources of the communication field. The complicated nature of organizations and the equivocality associated with ethical questions highlight communication as key to working through understandings of ethics in organizations [20]. Seeger [20] notes concerns such as advertising, deception, employee voice, management, and whistleblowing as representative issues addressed by organizational communication ethics, identifying communication to internal and external audiences as two broad domains of focus.

Prior to the emergence of explicit virtue ethics approaches, other perspectives on organizational communication ethics offered reflection and guidance for organizational participation, stemming from various perspectives loosely connected to the subfield of communication ethics and prompted by increasing concerns about organizational wrongdoing (e.g., [17,59]). Conrad’s [17] edited volume covered a broad range of issues relevant to ethics in organizational settings, a treatment prompted by the resurgence of interest in organizational values and a recognition of the complicated interrelationships among organizational culture, values, and relationships. Seeger [59] applied a Weickian enactment approach to understanding the role of values and ethics in a broad range of internal and external organizational processes. Seeger and colleagues [20,53,61] continued investigating ethics in organizations, particularly from an external stance. Seeger and Kuhn [62] reviewed ongoing research in organizational communication ethics, indicating its value as an area of investigation.

In the area of organizational or business ethics, much of the work on interpersonal communication ethics is identified with organizational justice or interactional justice, areas that have been studied from a communication perspective (e.g., [63,64,65]). However, Mainero and Jones [66] developed a communication ethics model of workplace romance based on the work of Jones [67], employing Rest’s [68] ethical decision-making model and drawing on some publications in the field of communication ethics, including Johannesen [69], Johannesen, Valde, and Whedbee [70], Neher and Sandin [71], Planalp and Fitness [72], and Stewart [73]. This study is an example of some strands of the field of communication ethics intersecting with the domain of business ethics in the area of personal workplace relationships, an intersection reaching back at least as far as the definitional attempt of Lewis [74], who, in an early study attempting to define business ethics, noted that “interpersonal communication is related to personal ethics in organization” ([74] p. 378). The next section reviews two major treatments of organizational communication ethics from a virtue perspective oriented toward the professions and the workplace that also includes a focus on workplace relationships to look toward a situated communication ethic for work/life relationships.

## 5. Reflections on a Situated Communication Ethic for Work/Life Relationships

Professional civility as applied to workplace relationships [8,13] is defined by communicative interaction that honors the organizational context as a “third”, to whom any organizational relationship is accountable. Relationships in organizations exhibit care for institutions [14] when the parties to those relationships order their interactions within the context of the organizational goods of productivity and organizational mission and subordinate relational goods to organizational ends. It is possible that the relationship may suffer harm or undergo reconfiguration as a result of this orientation. The potential clash of public and private domains is central to the framework of professional civility, which highlights the constraints of the work context that may come into conflict with expectations tied to the realm of personal friendship, as identified by Bridge and Baxter [21]. If both parties choose this approach to the ordering of goods, recognizing that the relationship may be compromised or diminished under these conditions, negotiating relational practices in the face of public/private sphere conflicts may result in less relational harm or the potential for reclamation, restoration, or recalibration in the future.

One element of the professional civility framework is the value of work as an end in itself, an element of professional ethics. This point resonates with the work of Cheney et al. [12] in their holistic approach to professional and organizational life. From Cheney et al.’s perspective, separating public and private is not possible, nor is it always desirable, because work is a key part of one’s identity. Considering the blended nature of workplace relationships raises the question of how to think about these issues and protect multiple goods at the same time, given the situated nature of much of workplace practice within organizational settings. A situated workplace communication ethic would take these issues into account.

Professional civility works within a virtue ethics framework. Virtue ethics, sometimes considered character-based ethics, focuses on what a good person would do when faced with an ethical challenge rather than referring to ethical principles to make a decision (in contrast to deontological or duty-based ethics) [75]. Virtue ethics can also be understood within the tradition of narrative ethics, which considers character to be defined and shaped by larger stories that identify the good for human life [76,77,78]. Virtues, in this perspective, emerge from narratives, larger stories or worldviews that provide sources of meaning for human life [15]. Cheney et al. [12] offer a helpful perspective, noting that ethics is “not just about specific decisions, but about entire ways of being and doing” ([12], p. 235). Rather than an atomized, rule-based approach to decision-making, this approach urges cultivating an ethical responsiveness based on thoughtful deliberation about the meaning of work and life. How those meanings manifest themselves will be shaped by an organization’s mission and by a commitment to work as a practice [8,9,10,11,12,13,14,15,16,17,18,19,20,21,22,23,24,25,26,27,28,29,30,31,32,33,34,35,36].

Organizational missions provide a narrative for employees [79]. During the organizational socialization process, employees learn the mission and values of the organization [5]. As employees take on an organizational identity, they will begin to enact the habits of the organization. These habits, which are tied to organizational culture, are rooted in the organization’s narrative [79]. In this sense, these habits become manifestations of organizational virtues of character, temporally situated inclinations, leanings, or dispositions giving rise to particular types of practices that cannot be specified in advance. From this perspective, workplace relationships orient within the mission structure of the organization, and their practices reflect organizational goods.

As organizational relationships form from patterned workplace interaction, personal and work-related domains of persons’ lives come into play. From both Fritz’s [8] and Cheney et al.’s [12] perspective, the navigation of these processes takes place within the thoughtful consideration of the larger organizational context, including the organizational mission and others in the workplace, the personal concerns of relational partners, and recognition of the influence of communication practices on others and the communication environment. This approach to workplace communication ethics is broad in scope, but points to the responsibility of participants to learn constructive communication practices relevant to their roles in the organization and to everyday interaction with others that transcends role responsibilities.

One example of a communication role responsibility is that of living the organizational mission. Supervisory behavioral consistency with an organization’s mission is associated with increased employee commitment [80,81]. This match of word and deed, known as managerial behavioral integrity, is an ethical practice for supervisors in relation to direct reports [82]. Whether the supervisor is involved in a close relationship with a direct report or not, this practice confirms and supports the organization’s mission and employees—the goods of place and people [8].

In the context of peer friendships, when one peer is promoted, each will face role-related tensions related to issues such as access to information and potential favoritism [21]. Considering the organization as a “third” to whom the relationship is accountable and owes care [14] would prompt the parties to the relationship to accept restrictions as to the type of information shared in the relationship and the need to make decisions on the basis of public criteria rather than on the basis of personal friendship. The ability to work within public roles and private roles would permit ethical behavior on the part of both parties. The context of the organization and the need to enact practices that work at a different level will shape the friendship, inevitably, but this new form will have its own integrity and identity.

Insights from work on positive communication speak to ethical communication in workplace relationships. Lutgen-Sandvik, Riforgiate, and Fletcher’s [47] study of supportive, positive communicative practices in the workplace provides insights into ways peers can support one another in the workplace. Acknowledging others’ contributions, celebrating successes, and finding opportunities to support coworkers creates a constructive work environment, enhancing the organizational environment and supporting productivity. This study, along with others, acknowledges the role of emotion in the workplace. This study also places problematic workplace relationships and interactions in stark contrast to what we know to be possible in thriving human communities gathered around shared goals.

A professional civility perspective would focus concerns about problematic workplace behaviors (e.g., bullying, workplace incivility, organizational retaliatory behavior, and destructive organizational behavior; see Section 2 of this article) on the particular area or areas of harm done by the behavior. Many, if not most, problematic behaviors, for instance, compromise productivity as well as the wellbeing of persons, and may also create a toxic workplace environment or climate. Problematic workplace behaviors that prevent, derail, or decrease the quality and quantity of work, create stress and poor experiences for others, and construct a negative, toxic workplace environment are unethical because of their deleterious effects on the goods of productivity (the work), place (the organization), and persons (others in the workplace). However, it is important to distinguish patterns of problematic behavior from legitimate and valuable differences in work styles that simply do not conform to our personal/cultural expectations, occasional bursts of frustration directed at others when one has had a bad day, and the inevitable transgressions we commit because we are imperfect human beings.

As Omdahl [83] points out, communicative processes of forgiveness are key to flourishing workplace relationships. When wrongs happen, conflict that may emerge in workplace friendships can jeopardize productivity [84]. Working within a situated ethic for workplace relationships would involve a determination to seek forgiveness [85] and/or a commitment to maintaining a professional distance that would allow the relationship to heal [86] or, at the very least, to permit work to continue even if the relationship is strained.

As is clear from this treatment, a situated communication ethic for workplace relationships would offer a comprehensive, holistic positioning of organizational involvement. This ethic defines an orientation to work life acknowledging the organization as a location with legitimate goals and aims whose purpose is to maintain itself [8]. At the same time, organizational participants are whole persons [12] who find meaning in work practices, whatever that work may be, and find much of that meaning in relationships with others in the workplace [3]. For those who have a more porous work/life boundary, close connections with peers or employees at other levels provide companionship, support, love, energy, and other goods in and beyond work life. An ordering of goods in work life makes it possible to honor organization, persons, relationships, and other organizational members, who may react in various ways to close connections of others in the workplace [3]. For persons who are not in close relationships in the workplace, the responsibility of attending to one’s work with cordial professional distance is a choice that permits meaning to emerge in different ways than those who seek connectedness at work.

## 6. Discussion

A robust approach to communication ethics in work/life relationships would build on current research and theory in each of these areas, taking account of existing and ongoing research in this area. Because much of the work on communication ethics in organizations is found in disparate locations, and because much of it is oriented toward the external domain (e.g., public relations, crisis communication), synthesizing the key themes and strands of scholarship relevant to workplace relationships would permit a synthetic approach that is situated within the workplace and takes organizational structure and constraints into account. The work of Horan et al. [3] provides an excellent foundation for examining outcomes across types of personal workplace relationships. Here, variables affecting the outcomes of these relationships permit an assessment of the effect of communication processes on persons, relationships, and organizations. The work of Fritz [50] on communication ethics in bullying relationships took this approach, focusing on outcomes and various ethical frameworks; similar work could be undertaken with work/life relationships within a more narrowly focused ethical framework derived from the considerations offered here.

A framework for a situated ethic of workplace relationships would draw on ongoing research on processes and practices in related areas, such as cultivating resilience in the workplace (e.g., [87]), particularly in an era where precarity is widespread and resources are scarce. In times of plenty, our focus of attention can turn to matters of less pressing urgency than the location of our next meal. In times of scarcity, stress and pressure may compromise our ability to attend to others in the workplace and prompt us to actions that in better circumstances we might not contemplate. In these circumstances, drawing on sources of meaning that call us to hope may give us opportunities to find our bearings again.

In an era moving toward a virtual or hybrid workplace, understanding the relational context of remote workers (e.g., [88]) for issues particular to their needs will open new avenues for investigation. Identifying practices that encourage productivity while supporting the good of relational connections in ways that reduce stress [89] will offer valuable insights informing ethical communication practices in remote relationships. This issue is likely to continue in salience as work practices shift in response to forms adopted during COVID-19.

Finally, a comprehensive approach to communication ethics in work/life relationships would acknowledge the reality of human variety and difference. The value of considering the workplace a public place rests in the recognition that we are in a moment of narrative and virtue contention regarding what is good for humans to be and to do [6]. Private convictions at variance with one another may become issues of contention in the workplace, preventing the formation of personal relationships. On the other hand, personal relationships formed around mutual liking and value similarity may promote the formation of cliques and the associated ostracism of dissimilar others [48,49]. Personal relationships that form, despite value differences, around a common commitment to work—a common center [40]—rather than on the basis of private value similarity or personal liking may prove to be durable and meaningful for the contemporary workplace. In the case of private friendship around value similarity and liking, ethical communication behavior honoring others and the organization might involve enacting practices that demonstrate distance between the close parties and invite excluded others into a space of conversation about work, generating task-focused bonds that permit common understanding and inclusion across levels of relational closeness. For personal relationships formed around a common center between parties with significant value differences, the solidity of connection on task- or mission-centered ground may allow a focus of attention on what the parties have in common beyond the workplace, despite variations in deeply-held political or religious beliefs. In this case, the flourishing of a work/life relationship is nourished by an organizational narrative that provides common ground for persons of difference.

## Data Availability

Not applicable.
